# A Study of Peripheral Arterial Disease in Type 2 Diabetes Mellitus Using the Ankle-Brachial Pressure Index and Its Correlation With Glycaemic Control and Duration of Diabetes in a Tertiary Care Teaching Hospital

**DOI:** 10.7759/cureus.90300

**Published:** 2025-08-17

**Authors:** Pranay Bandgar, Aniruddha S Jog, Vinayak Sawardekar, Smita Patil

**Affiliations:** 1 General Internal Medicine, Grant Government Medical College & Sir J J Group of Hospitals, Mumbai, IND; 2 Radiology, Grant Government Medical College & Sir J J Group of Hospitals, Mumbai, IND

**Keywords:** ankle-brachial pressure index, duration of diabetes, glycemic control, peripheral arterial disease, type 2 diabetes mellitus

## Abstract

Introduction: Peripheral arterial disease (PAD) is a macrovascular complication of diabetes mellitus. PAD is linked strongly with cardiovascular morbidity and mortality.

Objectives: To detect the burden of PAD in type 2 diabetes patients using the ankle-brachial pressure index (ABI) and to find its correlation with glycaemic control as well as duration of diabetes mellitus.

Methodology: This study was conducted on 300 type 2 diabetes mellitus patients aged 30-70 years, with diabetes for a minimum period of five years. All patients were screened for PAD by a vascular Doppler instrument using the ABI. Patients with ABI < 0.9 are subjected to colour Doppler to confirm the presence of PAD. Blood investigations, like fasting and postprandial blood glucose and glycated haemoglobin (HbA1C), were also done. Data were analysed by SPSS version 20 (IBM Corp., Armonk, NY) using appropriate tests.

Results: A total of 68 (22.7%) patients out of 300, who had diabetes mellitus for more than five years, had PAD. Prevalence increases with age and duration of diabetes. There was no difference between males and females. The possibility of PAD increases with raised body mass index (BMI). A total of 38 (55.9%) out of 68 patients with PAD were asymptomatic. Claudication was the most common symptom of PAD, found in 30 (44.1%) patients. Increased systolic pressure was found to be associated with PAD. The sensitivity and specificity of ABI in diagnosing PAD were found to be 89% and 94%, respectively. Chronic hyperglycaemia (high HbA1C) was found to be associated with a higher prevalence of PAD. ABI correlated significantly with the duration of diabetes, systolic hypertension, age, BMI, postprandial blood sugar level, and HbA1C.

Conclusion: Prevalence of PAD in diabetes mellitus is positively associated with duration of diabetes, age, BMI, systolic hypertension, and chronic hyperglycaemia (high HbA1C). It can be easily and reliably diagnosed using the ABI (less than 0.9), which will help in early detection and management.

## Introduction

Diabetes mellitus (DM) is a group of metabolic diseases characterised by hyperglycaemia resulting from defects in insulin secretion, insulin action, or both. The chronic hyperglycaemia of diabetes is associated with long-term damage, dysfunction, and failure of different organs, especially the eyes, kidneys, nerves, heart, and blood vessels [1]. According to a report by the International Diabetes Federation (IDF), approximately 537 million individuals globally were affected by diabetes, constituting roughly 10.5% of the world's population. This condition incurred healthcare expenditures totalling $966 billion. Projections indicate a surge in diabetes cases to 783 million by 2045, with associated healthcare costs estimated to surpass $1054 billion [2]. Diabetes-related complications affect many organ systems and are responsible for the majority of morbidity and mortality associated with the disease. Peripheral arterial disease (PAD) is a macrovascular complication. Cardiovascular disease (CVD) is also a major cause of morbidity and mortality for individuals with diabetes, and the largest contributor to the direct and indirect costs of diabetes [3]. PAD is linked strongly and independently with CVD morbidity and mortality, perhaps more strongly than prior myocardial infarction (MI). But PAD is less emphasised and less systematically evaluated than other atherosclerotic conditions or risk factors such as hyperlipidaemia and hypertension [4]. PAD is a progressive disorder characterised by stenosis and/or occlusion of large and medium-sized arteries, other than those that supply the heart (coronary artery disease, CAD) or the brain (cerebrovascular disease). There was a 36% prevalence of PAD among DM patients in India [5]. The treatment of patients with PAD can therefore be expensive, owing to the need for a variety of diagnostic tests, therapeutic procedures, and hospitalisations. Detection of asymptomatic PAD can reduce morbidity and financial loss due to PAD. It has additional value because it identifies patients at increased risk of atherosclerosis at other sites, and most importantly, CAD. The ankle-brachial pressure index (ABI) is a simple to perform, non-invasive, quantitative measurement of the patency of the lower extremity arterial system. It is very convenient to use in routine outpatient department (OPD) visits of diabetic patients. However, its reliability has not been studied to a great extent in the Indian population. Age, severity, and duration of DM are important predictors of PAD. However, very few studies have been conducted in Indian diabetic patients to find out the effect of duration and glycaemic control on the incidence and severity of PAD. Hence, we have conducted this study to detect the burden of PAD in type 2 diabetes mellitus (T2DM) patients using ABI and to find out its correlation with glycaemic control as well as duration of DM.

## Materials and methods

This tertiary care hospital-based cross-sectional study was conducted over a period of 21 months from January 2019 to September 2020. The study was approved by the institutional ethics committee under approval number IEC/PG/476/Dec/2018.

Study population

A total of 300 patients diagnosed with T2DM with a minimum duration of diabetes of five years were included from both outpatient and inpatient departments of the hospital.

Inclusion and exclusion criteria

Patients between 30 and 70 years of age were included in the study, independent of their gender. A history of T2DM for a minimum duration of five years was also one of the inclusion criteria. Patients with conditions that would interfere with the measurement of ABI were excluded. These were patients with trauma, surgery, or amputation involving the limbs, those with leg ulcers or deep vein thrombosis, and patients with filariasis or lower limb swelling due to other causes. People who were active smokers were also excluded. Females with gestational DM were not included in the study. Other patients who were excluded from the study included those with chronic kidney disease and patients on vasodilators or vasoconstrictors.

Data collection

After fulfilling the inclusion and exclusion criteria, patients were selected via simple random sampling. A detailed demographic and clinical history was collected using a case record form. This included age, sex, duration of DM, height and weight to calculate the body mass index (BMI), relevant past medical history, and presenting complaints. These complaints were graded according to Rutherford's classification [6]. Asymptomatic patients and mild claudication were considered in grade 0. Moderate to severe claudication was graded as grade I. Ischaemic rest pain was considered grade II, and any minor or major tissue loss was considered grade III.

All T2DM patients were screened by a vascular Doppler instrument for PAD using the ABI. Patients with an ABI <0.9 were subjected to colour Doppler of both lower limbs to confirm the presence of PAD [5]. Laboratory investigations performed included fasting and postprandial blood glucose and glycated haemoglobin (HbA1C) [7].

Data were analysed using SPSS version 20 (IBM Corp., Armonk, NY). All the continuous variables are presented as mean ± standard deviation (SD). The qualitative data are presented as proportions or ratios. The correlation is evaluated using the correlation coefficient. The rest of the results are presented using descriptive statistics. A p-value of less than 0.05 was considered statistically significant.

## Results

Out of a total of 300 patients who were included in the study, 68 had PAD. Therefore, the prevalence of PAD in patients with a duration of diabetes of five years is 22.7%.

Comparison of demographic characteristics in patients with and without PAD

The mean age of the study population was 53.3 ± 7.7 years. The mean age in the PAD group was 61.7 ± 5.0 years, while in the non-PAD group, it was 50.9 ± 6.5 years. In the age group-wise distribution, PAD was not seen in the age group below 50 years. In the 50-60 years group, it was seen in 22 (16.4%) out of 134 patients. In the above 60 age group, it was seen in 46 (65.7%) out of a total of 70 patients. Age distribution of PAD showed a significant difference between the groups (p = 0.001). A total of 184 males and 116 females were included, out of which 38 (20.6%) males and 30 (25.9%) females had PAD on the Doppler test. The difference in prevalence between males and females was not statistically significant (p = 0.294). Duration of diabetes was grouped as per the table below. We observed that, as the duration increased, the prevalence of PAD also increased. In patients with less than 10 years of diabetes, PAD was not found in any of the patients, while in those with a duration above 20 years, it was found in 32 (88.9%) out of 36 patients. BMI was compared for the prevalence. The average BMI for the total study group was 23.5 ± 1.9. In patients who had PAD, the average BMI was 25.3 ± 2, and in patients who did not have PAD, the average BMI was 23 ± 1.5. PAD was seen in 52 (47.7%) patients with a BMI above 24, as compared to 16 (8.4%) patients in the BMI below 24 group. The effect of BMI on PAD was statistically significant (p = 0.001) (Tables 1, 2).

**Table 1:** Comparison of demographic characteristics between patients with and without PAD. PAD: peripheral arterial disease.

Demographic characteristics		PAD	No PAD	Total
Number (n)		68 (22.7%)	232 (77.3%)	300 (100%)
Age		61.7 ± 5	50.9 ± 6.5	53.3 ± 7.7
Gender	Male	38 (20.6%)	146 (79.4%)	184 (100%)
	Female	30 (25.9%)	86 (74.1%)	116 (100%)
Duration in years		20.4 ± 4	9.3 ± 3.4	11.8 ± 5.8
BMI (kg/sq. m)		25.3 ± 2	23 ± 1.5	23.5 ± 1.9

**Table 2:** Demographic factors associated with PAD. * Age distribution, BMI, and duration of diabetes showed significant differences (p-value <0.05). PAD: peripheral arterial disease.

Demographic factors		PAD	No PAD	Total	Chi-square	P-value
Age group (years)	30-39	0 (0%)	10 (100%)	10 (100%)	105.1	0.001*
	40-49	0 (0%)	86 (100%)	86 (100%)		
	50-59	22 (16.4%)	112 (83.6%)	134 (100%)		
	60-70	46 (65.7%)	24 (34.3%)	70 (100%)		
Gender	Male	38 (20.7%)	146 (79.3%)	184 (100%)	1.102	0.294
	Female	30 (25.9%)	86 (74.1%)	116 (100%)		
Duration of diabetes (years)	Up to 10	0 (0%)	168 (100%)	168 (100%)	204.11	0.001*
	11-15	9 (14.5%)	53 (85.5%)	62 (100%)		
	16-20	27 (79.4%)	7 (20.6%)	34 (100%)		
	>20	32 (88.9%)	4 (11.1%)	36 (100%)		
BMI	Below 24	16 (8.4%)	175 (91.6%)	191 (100%)	61.23	0.001*
	Above 24	52 (47.7%)	57 (52.3%)	109 (100%)		
Total		68 (22.7%)	232 (77.3%)	300 (100%)	-	-

Presenting symptoms in patients

Claudication was the most common symptom reported in 30 (44.1%) patients. Claudication was graded from 0 to III. Asymptomatic patients were considered grade 0, and the presence of claudication indicated PAD. However, 38 (14.1%) patients amongst patients with claudication grade 0 were diagnosed with PAD. Therefore, despite having PAD, 38 (55.8%) out of 68 patients had no symptoms. Other symptoms in patients with PAD included rest pain, which was seen in 12 (17.6%) patients, paresthesia in 22 (32.4%) patients, and non-healing ulcers in eight (11.8%) patients. Systolic blood pressure readings were grouped as <140 mm Hg, 140-159 mm Hg, and 160 mm Hg and above. Prevalence was highest in the 160 mm Hg and above group, with 29 (74.4%) patients. When the prevalence of PAD in these blood pressure groups was compared, the difference between them was statistically significant (p-value = 0.001) on the chi-square test (Table 3).

**Table 3:** Clinical factors associated with PAD. * Claudication grade and SBP between the groups showed significant differences (p-value < 0.05). PAD: peripheral arterial disease; SBP: systolic blood pressure.

Clinical factors		PAD	No PAD	Total	Chi-square	P-value
Claudication grade	Grade 0	38 (14.1%)	232 (85.9%)	270 (100%)	113.7	0.001*
	Grade I	9 (100%)	0 (0%)	9 (100%)		
	Grade II	8 (100%)	0 (0%)	8 (100%)		
	Grade III	13 (100%)	0 (0%)	13 (100%)		
SBP	Less than 140	7 (4.4%)	152 (95.6%)	159 (100%)	85.033	0.001*
	140-159	32 (31.4%)	70 (68.6%)	102 (100%)		
	160-180	29 (74.4%)	10 (25.6%)	39 (100%)		

Claudication grades were compared with the grades of the ABI. In patients who had grade 0 claudication (asymptomatic), 247 (91.5%) patients had an ABI of 0.91 onwards. While 13 (54.2%) out of 24 patients with ABI between 0.6 and 0.79 had grade 3 claudication. The claudication grades, when compared with the ABI category, showed a statistically significant difference on the chi-square test (p = 0.001) (Table 4).

**Table 4:** Claudication and ABI category. * Claudication grade vs. ABI showed significant differences (p-value < 0.05). ABI: ankle-brachial pressure index.

Claudication grade	ABI (0.6-0.79)	ABI (0.8-0.90)	ABI (0.91 onwards)	Total	Chi-square	P-value
Grade 0	0 (0%)	23 (8.5%)	247 (91.5%)	270 (100%)	311.09	0.001*
Grade I	3 (33.3%)	6 (66.7%)	0 (0%)	9 (100%)		
Grade II	8 (100%)	0 (0%)	0 (0%)	8 (100%)		
Grade III	13 (100%)	0 (0%)	0 (0%)	13 (100%)		
Total	24 (8%)	29 (9.7%)	247 (82.3%)	300 (100%)		

Mean systolic pressure was higher in PAD patients, but diastolic pressure was almost equal. As expected, mean ankle pressure and ABI were lower in PAD patients as compared to non-PAD patients. The difference in systolic pressure, mean ankle pressure, and ABI was statistically significant between the two groups (p = 0.001) (Table 5).

**Table 5:** Comparison of mean SBP, DBP, ankle pressure, and ABI in patients with and without PAD. * The difference in SBP, ankle pressure, and ABI was significant between the groups (p-value < 0.05). ABI: ankle-brachial pressure index; PAD: peripheral arterial disease; SBP: systolic blood pressure; DBP: diastolic blood pressure.

	PAD	No PAD	Total	Value	ANOVA significance
Mean SBP (mm of Hg)	156.7 ± 12.1	134.1 ± 14.2	139.2 ± 18.7	142.039	0.001*
Mean DBP (mm of Hg)	77.6 ± 10.8	77.92 ± 10.5	77.9 ± 10.6	0.035	0.853
Mean ankle pressure (mm of Hg)	130.5 ± 11.8	140.6 ± 15.5	138.3 ± 15.3	24.547	0.001*
ABI	0.84 ± 0.08	1.05 ± 0.09	1.0 ± 0.13	306.1	0.001*

Clinical diagnosis using ABI was compared with the gold standard test of ultrasound Doppler. ABI less than 0.9 was considered positive. The performance of the ABI test was calculated using the above cut-off values. Its sensitivity was 89.7% and its specificity was 94%. The positive predictive value was 81.3%, and the negative predictive value was 96.9% (Table 6).

**Table 6:** Cross-tabulation of ABI vs. Doppler. ABI: ankle-brachial pressure index; PAD: peripheral arterial disease.

Clinical diagnosis	Doppler		Total
	PAD	No PAD	
Yes (ABI < 0.9)	61 (81.3%)	14 (18.7%)	75 (100%)
No (ABI > 0.9)	7 (3.1%)	218 (96.9%)	225 (100%)
Total	68 (22.7%)	232 (77.3%)	300 (100%)

Fasting and postprandial blood sugar levels were almost similar in the two groups. HbA1C value was greater in the PAD group. When compared using ANOVA, the difference in HbA1C between the PAD and non-PAD groups was statistically significant (p-value = 0.001). In patients who had HbA1C less than 7, only six (5.17%) patients had PAD. In patients who had HbA1C between 7 and 10, 37 (24%) patients had PAD, and 25 (83.3%) patients amongst those with HbA1C >10 had PAD. Therefore, good glycaemic control was associated with less PAD. The difference was significant, suggesting the effect of long-term glycaemic control on PAD (Table 7).

**Table 7:** Glycaemic control in the study population. * The difference in HbA1C was significant between the groups (p-value < 0.05). PAD: peripheral arterial disease; HbA1C: glycated haemoglobin.

Glycaemic control parameters	PAD	No PAD	Total	F-value (ANOVA)		P-value
Fasting blood sugar (mg/dL)	114.1 ± 22.3	114.4 ± 21.3	114.3 ± 21.5	0.010		0.920
Postprandial blood sugar (mg/dL)	187.1 ± 27.4	182.6 ± 27.9	183.6 ± 27.8	1.392		0.239
HbA1C (%)	9.76 ± 1.36	7.13 ± 0.9	7.72 ± 1.5	558.932		0.001*
HbA1C < 7 (good control)	6 (5.2%)	110 (94.8%)	116 (100%)	558.932	0.001*	
HbA1C = 7-10 (fair control)	37 (24%)	117 (76%)	154 (100%)			
HbA1C > 10 (poor control)	25 (83.3%)	5 (16.7%)	30 (100%)			

## Discussion

In this study, out of 300 patients, 68 patients had PAD and 232 were free of PAD. The prevalence of PAD was 22.6%. Diabetes is the most important risk factor for PAD, as seen in many studies. Agrawal et al. reported a prevalence of PAD of 28% in a study conducted among 11,157 participants [7]. Beks et al. observed a prevalence of PAD of 41.8% in diabetic patients [8]. Although investigators have found different values of prevalence, this can be explained by the differences in sample sizes, ethnic groups, and unidentified confounding factors.

The mean age of the study population, PAD, and non-PAD groups was53.3 ± 7.7, 61.6 ± 5, and 50. 8± 6.5 years, respectively. PAD was seen in the older age group, as it was found to be related to the duration of diabetes. PAD was not seen below 50 years in our study. The prevalence increased with age, and this correlation was significant (p = 0.001).

The mean age in most of the studies is about 50 to 60 years, which matches our study. A meta-analysis by Fowkes et al. showed increasing prevalence of PAD with age [9]. Adler et al. also observed age as a risk factor for PAD in diabetic patients [10].

Males and females having PAD were about 20% and 25% in the study population. PAD was not significantly related to gender distribution (p = 0.294). Fowkes et al., with a very large sample size in their study, also noted that there is no sex specific difference in PAD [9].However, some investigators, such as Akalu et al., have observed a significant preponderance of male patients in the prevalence of PAD [11].

The duration of diabetes had a significant impact on the prevalence (p = 0.001). As duration increased from 0 to 20 years and above, the percentage of patients diagnosed with PAD increased from 0% to around 88%. Most of the investigators have similar findings in their studies. According to a study done in Northwest India by Agrawal et al., with PVD, duration of diabetes was strongly associated with a regression coefficient (β) of 3.100 [7]. Hence, they concluded that the duration of type 2 diabetes is strongly associated with the risk of developing PAD.

Obese patients with a BMI higher than 24 were also found to be prone to PAD than those with a BMI less than 24 (p = 0.001). A study by Hicks et al. has revealed that a higher BMI increases the risk of developing PAD [12]. Some research has indicated that obesity may cause an inflammatory response in the body. The resulting inflammation may be the cause of PAD in obesity. Most of the investigators have noted the effect of obesity on PAD, but Akalu et al. did not find it significant. Small sample size (278) or inclusion of one particular ethnic group could be the reason for this discrepancy [11].

Claudication was the most common symptom (44.1%). However, even in PAD cases, 55.8% of patients were asymptomatic. Those having claudication grade I to III were found to have an ABI less than 0.9. Lower ABI values were correlated with more severe grades of claudication (p = 0.001).In one study, asymptomatic PAD was found to be 33% [13]. However, in this study, only ABI was used to diagnose PAD and not a Doppler study.

Mean systolic pressure (brachial) was greater, while ankle pressure and ABI were lower in PAD patients (p = 0.001). Diastolic pressures were similar in the two groups (p = 0.853). We also noticed that the poorer the systolic pressure control, the higher the prevalence of PAD (p = 0.001). In a study by Fowkes et al., the authors observed hypertension as a risk factor for the development of PAD with an odds ratio of 1.36 to 1.55 [9]. Adler et al. have also concluded in their prospective study of diabetes that a 10 mm Hg rise in systolic blood pressure is associated with 25% increased risk of PAD [10].

The performance of ABI was confirmed by the Doppler test. Sensitivity and specificity of ABI were 89.7% and 94%, with a cut-off value of 0.9 for ABI. In the PARTNERS study, most clinicians believed that the ABI was useful in the diagnosis and management of both symptomatic (96%) and asymptomatic (89%) PAD. Nearly all (88%) clinicians believed that it was feasible to incorporate ABI into daily practice. Overall, most clinicians (57-75%) believed that ABI was equal to, or more useful than, other widely available and reimbursed screening tests in preserving their patients' health. The study concluded that ABI is a simple PAD detection tool that can be successfully applied in primary care office practices [14]. Beks et al. in their study observed 20.9% of diabetic patients with ABI less than 0.9, while the actual prevalence of PAD was 41.8% [8].

Fasting sugar levels had no significant effect on prevalence (p > 0.05). HbA1C, which is considered the indicator of long-term glycaemic control, was significantly higher in the PAD group (p = 0.001). The poorer the long-term glycaemic control, the higher the prevalence of PAD. Similar findings were also noted by Adler et al. in their prospective diabetes study, and it was independent of other risk factors. In the same study, they observed that 1% rise in HbA1C is associated with a 28% increase in the risk of peripheral vascular disease [10]. In one meta-analysis, chronic hyperglycaemia (raised HbA1C) was related to macrovascular complications with a relative risk of 1.8 [15]. However, Beks et al. observed that HbA1C as well as fasting and postprandial sugar levels were significantly associated with PAD [8]. Correlation of age, BMI, systolic blood pressure, duration of diabetes, postprandial sugar level, and HbA1C was significant with ABI in our study. Similar observations are noted in other studies.

Limitations of the study

This study was done in a tertiary care teaching hospital in a metro city in India. This may not represent the complete demographic variability. This may have also led to selection bias, as patients presenting to the hospital may already have comorbidities in an advanced stage. Also, being a cross-sectional study, this provides just a one-time or snapshot relationship between PAD and its risk factors. It does not establish a temporal relationship or causal association. Although the sample size was 300, a modest sample size may limit the power to detect weaker associations. In addition, there is a possibility that the use of ABI for screening might give falsely recorded elevated readings in calcified and non-compressible arteries.

## Conclusions

The prevalence of PAD in DM is associated with the duration of diabetes, age, BMI, systolic hypertension, and chronic hyperglycaemia (HbA1C). It can be easily and reliably diagnosed using the ABI. ABI can be used as a simple and cost-effective screening tool to assess PAD. We have also found lower ABI values to be significantly associated with other factors such as advanced age and obesity. The prevalence of PAD is positively correlated with the duration of diabetes, indicating long-standing DM as one of the significant factors in the development of PAD. Chronic hyperglycaemia is associated with a higher prevalence of PAD, as evidenced by a positive correlation between HbA1C and PAD. The predictive value of point glucose measurements in assessing vascular risk is likely limited. The results of the study emphasise the importance of PAD screening in patients with diabetes of prolonged duration, in those with poor glycaemic control, and additional factors like obesity or older age. This highlights the multifactorial nature of the risk factors for PAD. Early diagnosis of PAD through ABI measurement can help in timely interventions like optimising diabetes control through lifestyle modification and pharmacotherapy, which may help in reducing the risk of this macrovascular complication.
